# The Role and Therapeutic Potential of Endothelial Progenitor Cells in Tumor Neovascularization

**DOI:** 10.1100/tsw.2010.100

**Published:** 2010-06-15

**Authors:** Branislava Janic, Ali S. Arbab

**Affiliations:** Cellular and Molecular Imaging Laboratory, Department of Radiology, Henry Ford Hospital, Detroit, MI, USA

**Keywords:** endothelial progenitor cells, vasculogenesis, angiogenesis, tumor, imaging probes

## Abstract

Although the cellular and molecular mechanisms of tumor growth and metastasis are not completely understood, it is established that formation and growth of new blood vessels is a conditio sine qua non for tumor survival, growth, and expansion. Numerous studies over the past decades demonstrated that neovascularization associated with tumor growth occurs via angiogenic and vasculogenic mechanisms that involve sprouting angiogenesis, intussusceptive angiogenesis, vessel co-option, vasculogenic mimicry, lymphangiogenesis, and the recruitment of endothelial progenitor cells (EPCs). Due to their ability to self-renew, circulate, home to the ischemic sites, and differentiate into mature endothelial cells, EPCs hold enormous potential to be used as a diagnostic and/or therapeutic agent in antitumor therapies. Hence, this review focuses on EPCs and their role in tumor angiogenesis with the emphasis on EPC recruitment/migration, and the potential use of EPCs as a therapeutic tool and imaging probe.

## INTRODUCTION

Tumor growth, invasion, and metastasis strongly depend on the development of new vascular networks that can supply sufficient amounts of oxygen and nutrients. This idea first came to light almost 50 years ago when Folkman and his group demonstrated that neovascularization is a necessary condition for malignant growth of solid tumors[1]. This pioneering work put forward the hypothesis that tumors are angiogenesis dependent and set the stage for the ever-since-expanding field of tumor angiogenesis.

Initially, tumor growth relies on diffusion of oxygen and nutrients from the surrounding tissue, without the need for a new blood supply. Under these conditions, a tumor can grow to a size of 1–2 $mm^3$. Thereafter, the growing metabolic demands associated with tumor growth are satisfied through growth and establishment of new blood vessels. To promote the needed neovascularization, tumor cells undergo the process known as “angiogenic switch”, where they acquire an angiogenic phenotype that changes the local equilibrium between positive and negative regulators of angiogenesis, and stimulates the formation of new vasculatures necessary for sustainable tumor growth[2,3,4,5]. Newly formed tumor blood vessels are usually leaky, dilated, and tortuous, and these structural and functional vessel abnormalities provide for hypoxic and acidic tumor microenvironment conditions that, in turn, further stimulate tumor angiogenesis, genetically select for more malignant cells, and contribute to the tumors' metastatic potential[6,7].

The formation of blood vessels occurs by two mechanisms: vasculogenesis and angiogenesis. Vasculogenesis is the process where blood vessels are formed de novo by *in situ* differentiation of the primitive progenitors, i.e., angioblasts, into mature endothelial cells and it was thought to take place during embryonic development only[8]. In contrast, angiogenesis occurs both during embryonic development and postnatal life, and is defined as a process that gives rise to new blood vessels by proliferation and migration of pre-existing, differentiated endothelial cells (ECs)[9,10]. It was generally considered that blood vessel formation during postnatal life was restricted to angiogenesis only and for decades, tumor vascularization was thought to be the exclusive result of the sprouting of new vessels from pre-existing vessels. However, recent studies demonstrated the existence of additional angiogenic and vasculogenic mechanisms associated with tumor growth, such as intussusceptive angiogenesis, vessel co-option, vasculogenic mimicry, lymphangiogenesis, and the recruitment of endothelial progenitor cells (EPCs)[11,12]. In most cases, these mechanisms take place concomitantly and are the potential targets for novel antiangiogenic/antitumor therapeutic strategies. This review focuses on EPCs and their role in tumor angiogenesis, with the emphasis on EPC recruitment/migration relevant to tumors, and the potential use of EPCs in novel therapies and as imaging probes to differentiate disease conditions.

## ENDOTHELIAL PROGENITOR CELLS

A growing body of evidence indicates that neovascularization processes associated with tumor growth are in part supported by recruitment of bone marrow–derived, endogenous EPCs and their functional incorporation into the new vasculatures. Previous studies demonstrated the existence of circulating ECs in peripheral blood in various vascular diseases[13,14,15,16,17]. However, for a while it was unclear whether these cells or their precursors played a role in postnatal vascular growth. The breakthrough came from the work by Asahara and Murohara[18] who demonstrated the presence of CD34+/vascular endothelial growth factor receptor-2 (VEGFR2) + EPCs in human peripheral blood. These cells gave rise to mature ECs in culture and were capable of incorporating into the sites of active neovascularization in animal models. This landmark work opened the possibility that in adults, endothelial stem or precursor cells may contribute to the formation of new blood vessels by vasculogenesis. Since then, researchers have been gaining significant insights into the postnatal neovascularization and the origin, phenotype, and function of the EPCs. Studies have implicated EPCs to play a critical role in adult, postnatal endothelial repair and vasculogenesis that accompanies physiological and pathological conditions, such as myocardial ischemia and infarction, limb ischemia, wound healing, atherosclerosis, endogenous endothelial repair, and tumor vascularization[19]. However, the main factor hindering EPC research is the controversy on the identity of EPCs. Earlier studies defined EPCs as the cells coexpressing hematopoietic stem cell marker CD34 and endothelial marker VEGFR2[20]. Since subsequent work showed that some mature ECs also coexpress CD34 and VEGFR2, and that CD34 was not an exclusive marker for hematopoietic cells, a novel CD133 glycoprotein was accepted as a more appropriate marker for immature progenitor cells[21,22]. The glycosylated form of CD133 protein is expressed on hematopoietic stem cells, but not on mature ECs, and it is recognized by AC133 monoclonal antibody[23,24]. Rafii and colleagues suggested that a subset of circulating CD34+ cells that are positive for both VEGFR-2 and AC133 represent a functional EPC population that plays a role in postnatal angiogenesis or vasculogenesis[22]. EPCs also share many cell surface markers with ECs and with stem/progenitor cells of different tissues[25]. However, it is currently customary to define EPCs as cells that are positive for AC133, CD34, and VEGFR2 markers, with the following distinction: AC133+/CD34+/VEGFR2+ cells represent an immature, highly proliferative EPC population localized mainly in the bone marrow, while AC133-/CD34+/VEGFR2+ cells are considered circulatory, more mature cells that are limited in their proliferative capacity[19]. In addition, these more mature cells also express some of the endothelial-specific antigens, such as platelet endothelial cell adhesion molecule 1 (PECAM-1 or CD31), E-selectin (CD62E), VE-cadherin (CD144), and chemokine receptor CXCR-4 (CD184), and have the ability to migrate in response to the CXCR-4 ligand, stromal derived factor-1 (SDF-1), and vascular endothelial growth factor (VEGF). It is now generally accepted that new vessels can also be formed *via* recruitment of circulating EPCs. Indeed, studies in recent years demonstrated that intravenously administered progenitors isolated from bone marrow, peripheral blood, or cord blood can home to ischemic sites, including tumor sites, emphasizing the significance of the paracrine effect of tumor-secreted factors. Despite the significant amount of data available, controversy still remains on the identity and function of the putative EPC and its functional significance and contribution to tumor vasculature and growth. Gradually, the consensus on the putative EPC phenotype is arising; nevertheless, further identification and characterization of novel, more specific, EPC markers is warranted.

## THE ROLE OF EPCS IN TUMOR ANGIOGENESIS

Mobilization of EPCs from the bone marrow (BM) into the circulation, their homing to the sites of neovascularization, and subsequent differentiation into mature ECs are tightly regulated processes. Investigators have tried to identify the paracrine signals associated with tissue ischemia and tumor growth that create a favorable environment that attracts EPCs. The recruitment of EPCs from BM is initiated by increased circulatory levels of factors, such as VEGF, FGF, SDF-1, GM-CSF, osteopontin, etc., that are released by growing tumors. These factors activate the BM microenvironment to switch from a dormant to a proangiogenic state and the process involves the activation of matrix metalloproteinase-9 that releases the BM stromal cells' membrane bound c-Kit (CD117) ligand. The generated soluble form of the c-Kit ligand stimulates c-Kit–positive EPCs to move from the BM niche to the BM vascular zone and translocate to the circulation[26]. Tissue hypoxia present in tumors and ischemic vascular diseases is considered to be central to this paracrine mechanism and this ischemic effect was shown to be mediated by a marked increase in VEGF and SDF-1 circulating levels[3,27,28]. VEGF and SDF-1 expressions are transcriptionally up-regulated by tissue hypoxia–induced expression and/or activation of hypoxia inducible factor 1 (HIF-1)[28,29,30]. In addition to the increase in their circulatory levels, VEGF and SDF-1 expressions are increased locally, within the hypoxic tissue itself, which in turn stimulates recruitment of progenitor cells to the hypoxic site as well[31,32]. Our recent work also indicated that the homing of EPCs into the neovessels of implanted tumors was related to HIF-1$\alpha$–induced SDF-1 expression[33]. Using rat glioma and melanoma models, we traced the migration of locally implanted, magnetically labeled, cord blood–derived AC133+ EPCs. After completely mixing two populations of cells (AC133+ and tumor cells) and implanting them as a mixture, one would expect a homogenous distribution of human AC133+ cells throughout the growing tumor mass. However, after implantation, AC133+ cells redistributed and when the tumor grew to 1–1.5 cm in size, MRI and histological analysis revealed that most of the cells migrated towards the peripheral parts of the tumor that colocalized with the strong expression of HIF-1$\alpha$ and SDF-1, which indicated the more hypoxic microenvironment (Fig. 1). The same areas also expressed high levels of PDGF and MMP-2 that usually reflects high angiogenic activity. We also showed by immunofluorescent staining that human AC133+ cells that were detected at the periphery incorporated into the tumor neovasculature (Fig. 2)[33].

In addition to shedding more light onto the biological mechanisms of EPC migration within the tumor environment, this work also introduced a novel noninvasive MRI method for *in vivo* stem cell tracking that may facilitate the development of novel diagnostic and treatment strategies. Recent studies also demonstrated the correlation between VEGF and SDF-1 expression at the transcriptional and functional/effector level. VEGF was shown to up-regulate SDF-1 and CXCR-4 molecules[34,35]. In addition, Kollet et al. demonstrated the possible synergistic effect between two cytokines by showing that without a concurrent VEGF signal, SDF-1 was insufficient in recruiting EPCs to tumor sites[36]. In addition to VEGF and SDF-1, numerous other factors produced by tumor and surrounding cells have been

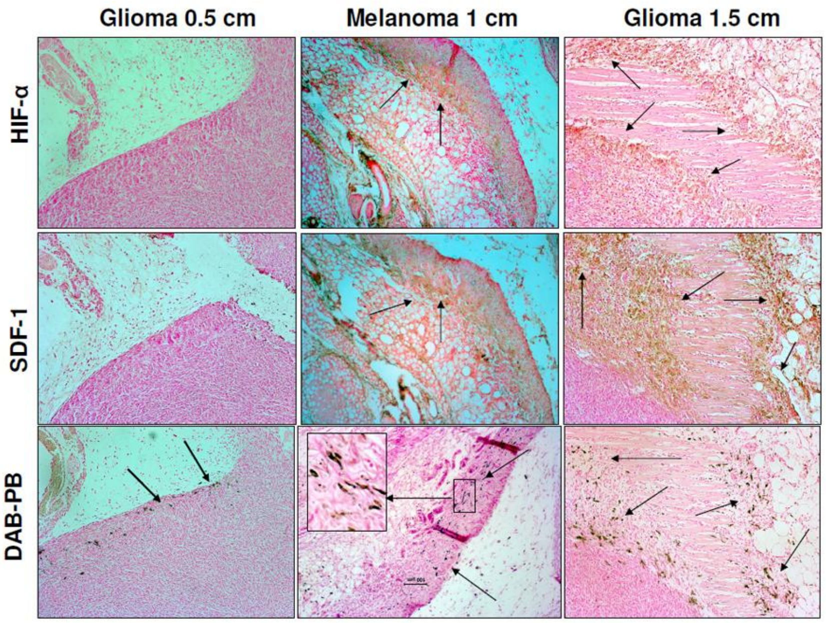

**FIGURE 1.** Expression of HIF-1α and SDF-1 at the sites of migrated labeled AC133+ cells in tumors. Left column: Expression of HIF-1α and SDF-1 at the sites of migrated labeled AC133+ cells in rat glioma at tumor size of 0.5 cm (consecutive sections). Prussian blue staining shows migration of cells at the periphery of the tumor (arrows). HIF-1α and SDF-1 staining do not show expression of the factors at the corresponding sites of migrated cells. All images are of 10× magnification. Middle column: Expression of HIF-1α and SDF-1 at the sites of migrated labeled AC133+ cells in human melanoma at tumor size of 1 cm (consecutive sections). Prussian blue staining shows migration of cells at the periphery of the tumor and at the sites of invasion into surround muscles and tissues (arrows). HIF-1α and SDF-1 staining show very strong localized expression of the factors at the corresponding sites of migrated cells (arrows). All images are of 10× magnification. Right column: Expression of HIF-1α and SDF-1 at the sites of migrated labeled AC133+ cells in rat glioma at tumor size of 1.5 cm (consecutive sections). Prussian blue staining shows migration of cells at the periphery of the tumor and at the sites of invasion into surround muscles and tissues (arrows). HIF-1α and SDF-1 staining show very strong localized expression of the factors at the corresponding sites of migrated cells (arrows). All images are of 10× magnification.

implicated to play a role in EPC recruitment to tumor sites. Tumor-produced CCL2 and CCL5 were reported to mobilize EPCs from the circulation[37]. Hormones such as 17-b-estradiol were shown to stimulate EPC-dependent neovascularization[38]. Neurotrophin-induced angiogenesis was implicated in breast tumor growth[39,40] and in ischemic animal models[41]. Adiponectin, a peptide hormone secreted by adipocytes, was also shown to promote EPC numbers, migration, and mammary tumor growth in animal models[42,43,44]. In addition, factors that regulate physiological angiogenesis can play a role in EPC recruitment and mobilization. Many of the described paracrine factors that are involved in EPC recruitment have been the target of novel antiangiogenic therapies. However, to be able to develop more specific strategies, the exact molecular mechanisms responsible for EPC mobilization and homing to tumor sites still need to be dissected.

It is hypothesized that once recruited to tumor sites, EPCs can have a dual role in tumor angiogenesis, i.e., they can provide structural function by incorporating into the vessels and a supporting paracrine role where they secrete angiogenic factors[45]. Our initial results with cord blood–derived EPCs also showed both synergistic as well as paracrine effects in the formation of tube-like structures in the matrigel plate by microvascular endothelial cells (MEC)[46]. Many attempts have been made to answer: What is the actual contribution of EPCs to tumor vessel growth and, more importantly, do EPCs incorporate into the newly formed vasculature? One of the first demonstrations of the EPC contribution to tumor neovascularization

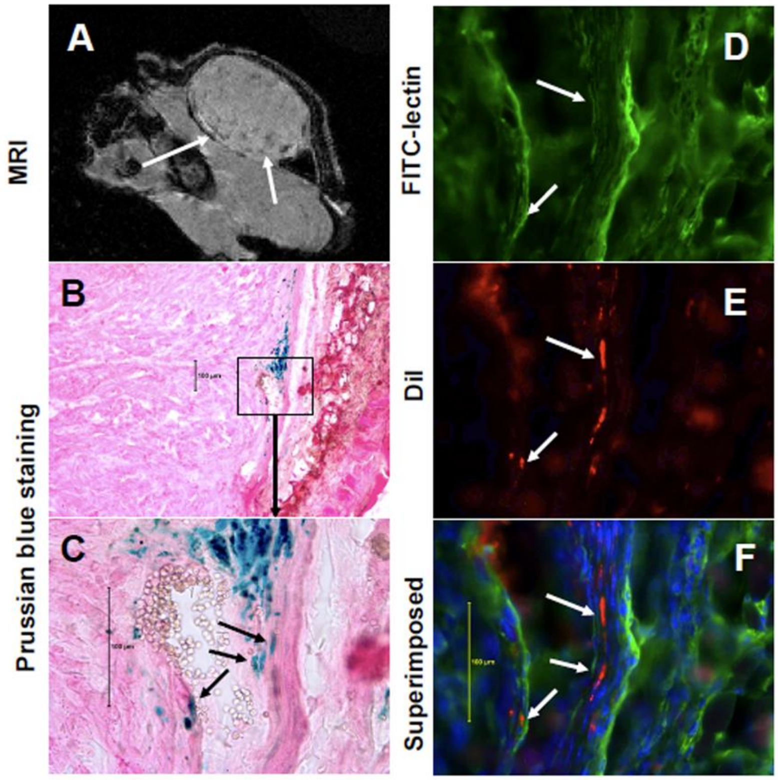

**FIGURE 2.** Migration and incorporation of locally administered magnetically labeled AC133+ cells in human melanoma tumor in mouse. (A) Gradient echo MR image shows low signal intensity areas mostly at the periphery of the tumors (white arrows). (B,C) Representative histology sections show incorporation of Prussian blue–positive cells along the margin of a blood vessel (note the RBC within the lumen) (Prussian blue staining was performed after obtaining fluorescent microscopic images, please see below). (D,E,F) To prove whether implanted live FePro-labeled AC133+ cells incorporated in the formation of tumor angiogenesis (vasculature), tumor vasculature (endothelial lining) was delineated by FITC-labeled tomato lectin, and fluorescent microscopic images were obtained both at FITC (lectin) and rhodamine (DiI-labeled cells) channels. Cell nuclei were delineated by DAPI staining. After the fluorescent microscopy, same sections were stained with Prussian blue (for iron-positive cells) and bright-field microscopic images were obtained to match the area of fluorescent-positive cells. Fluorescent microscopic images show incorporation of locally implanted DiI-labeled AC133+ cells (white arrows) into the tumor vasculatures. Arrows indicate lectin-positive endothelial lining and DiI-positive AC133+ cells. (Reproduced from Arbab, A.S. et al. [2008] *FASEB J.* **22,** 3234–3246; with permission).

was by Lyden et al.[47], where they showed that transplantation of circulatory EPCs restored tumor angiogenesis and growth in an angiogenesis-defective, Id1+/-Id3-/- host mouse, and donor-derived cells were detected throughout the tumor neovessels, with 90% percent of contribution. Since then, many similar studies were done, however, the reported percentages of EPCs contributing to the tumor neovascularization varied significantly depending on the tumor model used. Recent human studies on patients with non–small cell lung cancer and lymphoma showed increased numbers of AC133+ and/or EPCs in the endothelial tubes of tumor capillaries[48,49,50]. The mean contribution of EPCs to human tumor vasculature reported from transplantation study ranged from 1% (head and neck sarcoma) to 12% (lymphoma)[51,52]. On the other hand, Purhonen et al.[53] reported that in animal models, BM-derived or other EC precursors did not contribute to tumor vascular endothelium at all, and that cancer growth does not require BM-derived endothelial progenitors. Since demonstrating the significant luminal incorporation of EPCs within the tumor neovasculatures proved very challenging, the biological role of

EPCs in tumor angiogenesis was very often questioned. On the other hand, more recent reports showed that specific ablation of BM-derived EPCs with anti-VE-cadherin antibody markedly impaired tumor growth associated with reduced vascularization[54,55]. This also supports the idea that even with the low vessel incorporation rate, the pararcrine EPC function may be the one that is critical for tumor angiogenesis. Nolan et al.[54] also indicated that the percentage of incorporated BM progenitors and the density of BM progenitor–derived vessels might depend on the stage and size of the tumor. Therefore, the differences in EPC incorporation in previously published reports may not only be due the diversity of tumor models/types studied, but also due to the temporal differences in tumor development at the time of the study. To be able to develop precise EPC-targeted antiangiogenic therapy, the complete understanding of temporal and spatial EPC distribution in relation to tumor growth/size is extremely important. The work done by our group analyzed this relationship by tracking the migration and vascular incorporation of magnetically labeled human AC133+ cells in animal models[33,56]. In a mouse glioma model, magnetically labeled cells were intravenously administered to the tumor-bearing animals at the time of tumor implantation or after tumors grew to 0.2 cm in size. Labeled EPCs were detected by MRI within 3–5 days in tumors that were established prior to EPC administration, and within 5–7 days in animals where tumors and EPCs were concurrently implanted. At this early stage of tumor growth (0.5–1 cm), MRI analysis revealed incorporation of labeled AC133+ cells into tumors mostly along the tumor margins, i.e., periphery at the tumor, which was confirmed by Prussian blue histological staining (Fig. 3). However, once the tumor reached the size of 1 cm, differences in the timing of labeled cells administration were no longer reflected on MRI and histopathology images. Although not quantified, vascular incorporation of administered cells was clearly demonstrated by immunofluorescent staining (Fig. 4). At the later stages of tumor growth, iron-positive cells detected on MRI and by Prussian blue were no longer at the periphery of the tumor, and as previously suggested[54], it is possible that at later stages of tumor development, administered EPC-derived vessels are diluted with non-EPC–derived vessels from the periphery of the tumor.

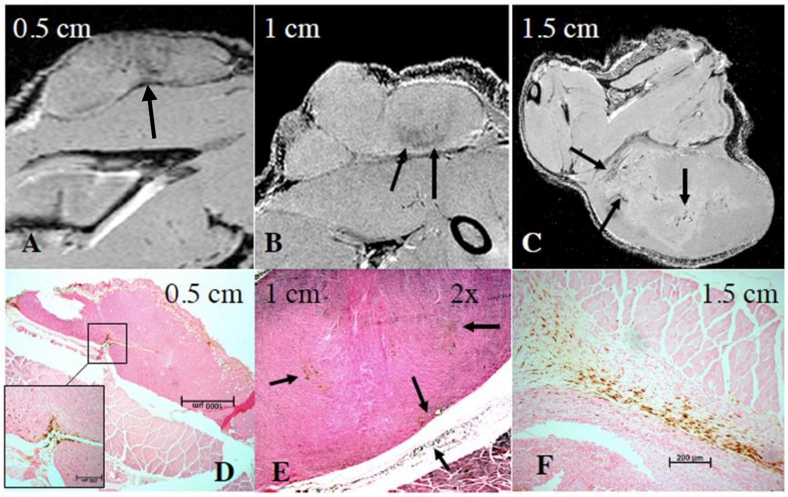

**FIGURE 3.** *Ex vivo* (A–C) MRI of flank tumors at different stages of development and corresponding histology with Prussian blue staining (D–F). Labeled human AC133 cells were injected 3 days after tumor implantation into immunodeficient mice. Tumors at the sizes of 1 and 1.5 cm show areas of low signal intensity on *ex vivo* MRI due to the presence of migrated labeled AC133+ cells (arrows). Prussian blue staining (DAB-enhanced) shows iron-positive cells at the periphery of the tumors. Scale bars 1000 µm (D), 200 µm (F).

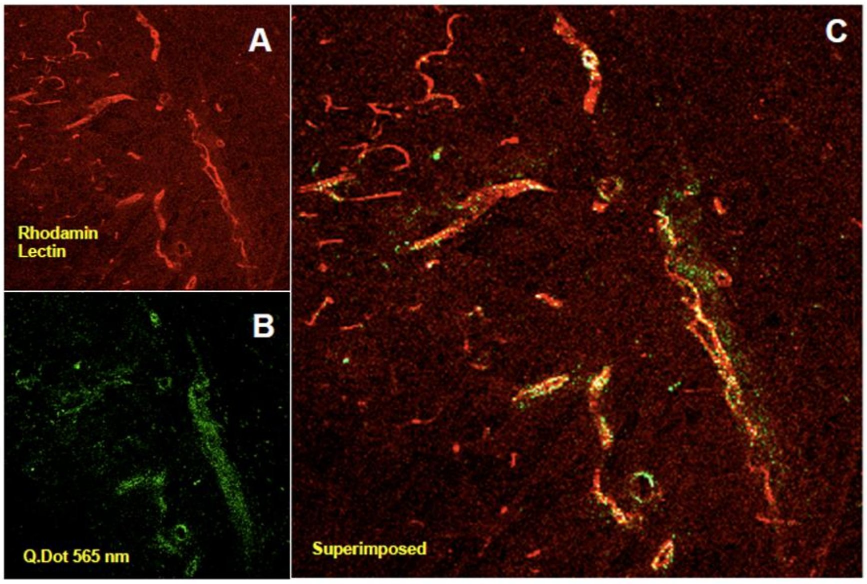

**FIGURE 4.** Incorporation of intravenously injected human AC133 cells into the vasculature of the implanted tumor. Vessels are delineated by rhodamine-labeled lectin (red) and the quantum dot–labeled ECs show fluorescent green. (A–C) Confocal microscopic images. (A) Tumor vessels delineated by rhodamine-labeled lectin. (B) Quantum dot–positive cells (green). (C) Superimposed images.

Regardless of the variable success among the studies in showing the significant EPC contribution to tumor neovasculatures, the majority of human studies are in agreement with respect to the increase in numbers of circulating EPCs in tumor pathologies. Increased frequencies of EPCs in peripheral blood were found in patients with malignant gliomas[57,58], non–small lung cancer[49], myeloid metaplasia[59], myeloid leukemia[60], hepatocellular carcinoma[61], colorectal cancer[62], myeloma multiplex[63], lymphoma[50], and breast cancer[64,65]. Therefore, EPCs have the potential to serve as a novel biomarker in diagnosing the disease, designing therapies, and assessing the prognosis in cancer patients.

## EPCs AS THERAPEUTIC AND IMAGING TOOL

An increasing body of evidence showing that EPCs are recruited to ischemic sites and tumor vasculatures opens the venue for novel antitumor strategies. One of the therapeutic approaches that can be developed to treat tumors is to use these “tumor-homing” cells as a therapeutic delivery vehicle that could promote antiangiogenic and/or cytotoxic effects. Treatment strategies that gained significance during the past decades utilize viral vectors carrying various cytokine, suicide, or other oncolytic genes. One of the greatest challenges of vector development in the current gene therapy approaches is targeting a therapeutic gene to diseased cells with the aim of achieving sufficient gene expression in the affected tissue, while minimizing toxicity and expression in other tissues. To overcome these limitations, cell delivery systems utilizing various types of cells have become the focus of many investigations and, due to their unique property to migrate to pathological lesions, stem cells are considered to be the vehicle of choice for gene delivery to tumors[66,67]. Since they exhibited active migration and incorporation into the tumor neovasculatures when administered locally or systemically[56,68,69,70], AC133+ EPCs, as a subpopulation of pluripotent hematopoietic stem cells (HSC), have also been studied as a potential gene delivery system. Ferrari et al.[71] have shown the migration and incorporation of retrovirally, HSV-tk–transfected mouse EPCs in a subcutaneous glioblastoma mouse model. Interestingly, due to their homing properties, EPCs were pointed out as a favorable gene delivery vehicle when replication-competent viruses were considered[72] and proof of the concept of targeted retrovirus delivery was reported by Jevremovic et al.[73], demonstrating that endothelial lineage progenitors could successfully and specifically transfer retrovirus to tumor cells in mice. However, the successful clinical application of future EPC transplantation-based therapies is limited by lack of the adequate, noninvasive imaging approaches for monitoring the fate and tissue distribution of transplanted progenitors. We have investigated the feasibility of using cord blood–derived EPCs as both gene carriers and imaging probes in breast cancer animal models. We used magnetically labeled EPCs to carry the human sodium symporter gene (hNIS) to the sites of implanted breast cancer in a mouse model. We utilized MRI and single photon emission computed tomography (SPECT) as noninvasive imaging modalities to monitor the fate of administered cells. MRI analysis revealed the accumulation of magnetically labeled, intravenously administered EPCs, and SPECT confirmed the transgenic expression of functional hNIS protein at the tumor site (Fig. 5)[74]. Based on the characteristics of EPCs, including their self-renewal ability, these cells indeed may be a convenient reagent for *ex vivo* expansion and manipulation that generate cellular imaging probes. These cells can be *in vitro* labeled with MRI imaging contrast agents, such as iron oxides or gadolinium, or they can be genetically modified to express genes that are used as probes in nuclear medicine or optical imaging. Our ongoing current work is to explore the value of using EPCs as imaging probes to differentiate glioma from radiation necrosis based on the presence of active angiogenesis. The distinguishing characteristic between recurrent glioma and radiation necrosis is that there is very little active angiogenesis at the site of radiation necrosis[75]. If the site of active angiogenesis can be determined by *in vivo* imaging, recurrent glioma could easily be differentiated from radiation necrosis. Our initial results are encouraging and will be published in due course. Similarly, *ex vivo*–expanded EPCs can be used to determine the therapeutic effects for the treatment of ischemia or infarction by using EPCs as imaging probes.

Overall, while taking advantage of the given, favorable, i.e., hypoxic, microenvironment that can guide EPCs to the tumor site by homing signals, as well as the inherent potential of EPCs to stimulate neovascularization, systemic or local administration of allogeneic or autologous stem cells may be utilized for targeted delivery of the gene of interest to tumor sites. However, lack of agreement on the EPC phenotype, as well as the limited quantities of EPCs that can be obtained from the patient, have been major factors impeding the effective development of such therapies. Therefore, further studies and consensus are needed concerning the appropriate procedures for isolating, potentiating, and phenotyping of EPCs. In addition, given the variability in neovascularization mechanisms and the relative EPC role associated with different tumor types and stages, further investigation is absolute in order to deliver EPC-based gene therapy–mediated antiangiogenic/antitumor therapy. In addition to being a gene carrier, EPCs can also serve as targets for therapy. Reducing the EPC mobilization and/or pararcrine angiogenic effect may serve as an alternative antiangiogenic adjuvant therapy that may enhance the already ongoing cytotoxic antitumor regimen. Indeed, it was reported that transient disruption of the SDF-1/CXCR4 axis using CXCR4 blocking antibody blocked the recruitment of bone marrow–derived cells into the tumor tissue, and resulted in complete inhibition of accelerated tumor growth after chemotherapy in mouse[76]. However, to further pursue EPCs as a target for antitumor strategies, future investigations are needed to determine tumor-specific patterns of EPC mobilization and migration, as well as to understand the extent and molecular mechanism of EPC contribution to tumor neovasculature formation, growth, and maintenance.

In a summary, a significant amount of evidence supports the fact that bone marrow–derived EPCs play an important role in tumor vascularization and growth. Although the mechanisms and the magnitude of EPC involvement in tumor-associated angiogenic and vasculogenic processes are not certain, these cells present themselves as a valuable tool for designing future antitumor strategies. The particular advantage when creating an EPC-based therapeutic approach comes with the prospect that these cells can be used as therapy targets, gene/protein delivery vehicles, and imaging probes.

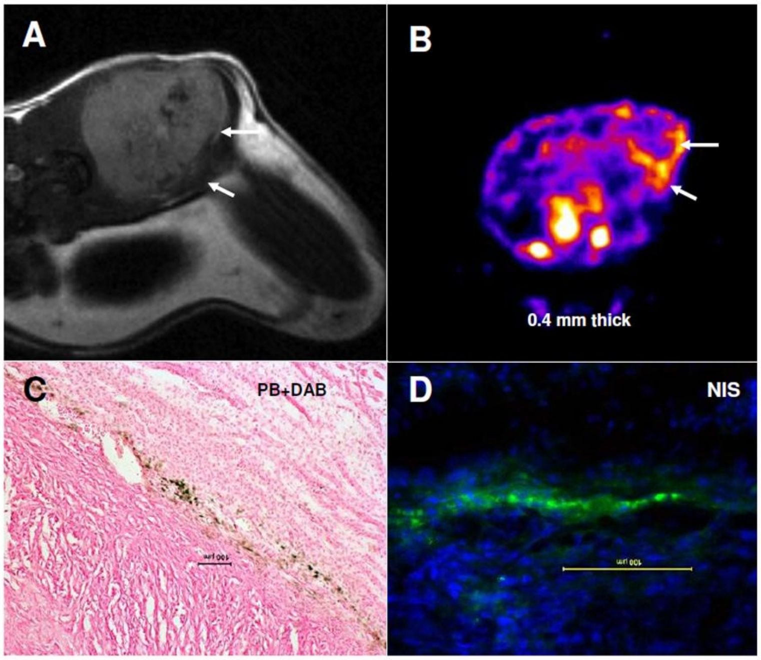

**FIGURE 5.** Accumulation of magnetically labeled, transgenic AC133+ progenitor cells around the implanted tumor. MRI shows low signal intensity areas at the margin of the tumor (A), which are at the corresponding sites of iron-positive cells detected by Prussian blue staining (C). The central low signal intensity areas are due to hemorrhagic foci within the tumor. Transaxial sections of SPECT study (B) indicate the accumulated transgenic AC133+ cells that are detected by T-99m. The SPECT study also proves the migration and homing of AC133+ cells at the margin of the tumors (seen on MRI). Immunohistochemistry shows the accumulation of hNIS-positive cells at the corresponding sites, as detected by labeled secondary antibodies (D). The findings prove that AC133+ EPCs can carry reporter or therapeutic genes to the site of interest (here at the site of active angiogenesis) and magnetically labeled AC133+ EPCs can act as probes for cellular MRI.

## ACKNOWLEDGMENT

This work was supported by NIH grants R01CA122031 and 1R21CA129801, and AHA grant 09SDG2230011.
